# Correction to “One‐Step Non‐Invasive Diagnosis of Metabolic Dysfunction‐Associated Steatohepatitis and Fibrosis in High‐Risk Population”

**DOI:** 10.1002/ueg2.70318

**Published:** 2026-11-02

P. Iruzubieta, R. Mayo, I. Mincholé, et al., “One‐Step Non‐Invasive Diagnosis of Metabolic Dysfunction‐Associated Steatohepatitis and Fibrosis in High‐Risk Population,” *United European Gastroenterology Journal* 12, no. 7 (2024): 919–929, https://doi.org/10.1002/ueg2.12589.

In the originally published article, the colors of the stacked bars in Figure 1 did not correspond to the diagnostic categories indicated in the color key. The correct color coding is green for isolated steatosis, yellow for early MASH, and red for at‐risk MASH. Accordingly, the green and red colors in all stacked bars of Figure 1 should be interchanged, while the yellow color and the existing color key remain unchanged.

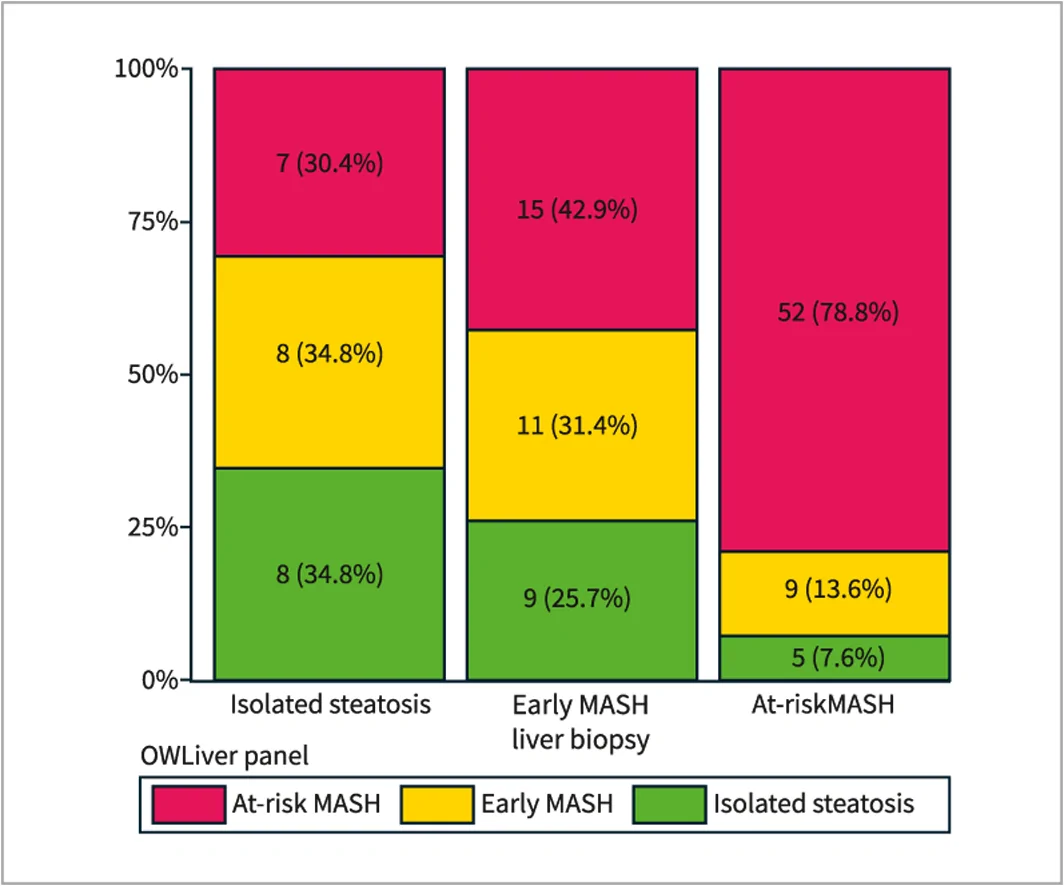

The numerical values and percentages shown in Figure 1 are correct. This correction does not affect the analyses, results, or conclusions of the article.

We apologize for this error.

